# Supplementary material for: A nonparametric alternative to the Cochran-Armitage trend test in genetic case-control association studies: The Jonckheere-Terpstra trend test
Source: PLoS One. 2023 Feb 2;18(2):e0280809. doi: 10.1371/journal.pone.0280809 (PMC9894441; doi:10.1371/journal.pone.0280809)
Supplement: S1 Table — (DOCX) [file pone.0280809.s001.docx]

## Table S1: Power comparison between $\boldsymbol{T}_{\boldsymbol{JT}}$ and $\boldsymbol{T}_{\boldsymbol{CA}}^{\boldsymbol{Add}}$ for the additional simulation settings ($\boldsymbol{q=0.4}$)

|  |  | Statistical Power | |
| --- | --- | --- | --- |
| N | Genetic model ($\theta/\pi$) | $T_{JT}$ | $T_{CA}^{Add}$ |
| 1500 | 0.25 (Dominant) | 0.7751 | 0.7392 |
| 1500 | 0.30 | 0.7943 | 0.7766 |
| 1500 | 0.3524 (Additive) | 0.8048 | 0.8019 |
| 1500 | 0.45 | 0.7915 | 0.8031 |
| 1500 | 0.45 | 0.7631 | 0.7934 |
| 1500 | 0.50 (Recessive) | 0.7036 | 0.7635 |
| 2000 | 0.25 (Dominant) | 0.8812 | 0.8518 |
| 2000 | 0.03 | 0.9026 | 0.8895 |
| 2000 | 0.3524 (Additive) | 0.9105 | 0.9082 |
| 2000 | 0.40 | 0.8934 | 0.9029 |
| 2000 | 0.45 | 0.8758 | 0.8978 |
| 2000 | 0.50 (Recessive) | 0.8266 | 0.8696 |
