# Supplementary material for: A nonparametric alternative to the Cochran-Armitage trend test in genetic case-control association studies: The Jonckheere-Terpstra trend test
Source: PLoS One. 2023 Feb 2;18(2):e0280809. doi: 10.1371/journal.pone.0280809 (PMC9894441; doi:10.1371/journal.pone.0280809)
Supplement: S2 Table — (DOCX) [file pone.0280809.s002.docx]

**Table S2: Comparison between the Jonckheere-Terpstra trend test (**$\boldsymbol{T}_{\boldsymbol{JT}}$**) and the Cochran-Armitage trend test (**$\boldsymbol{T}_{\boldsymbol{CA}}^{\boldsymbol{Add}}$**) on SNPs that were reported associated with hypertension***

| SNP | $r_{0}$ | $r_{1}$ | $r_{2}$ | $s_{0}$ | $s_{1}$ | $s_{2}$ | $T_{JT}$ | $T_{CA}^{Add}$ | $\Delta T$ | Model |
| --- | --- | --- | --- | --- | --- | --- | --- | --- | --- | --- |
| rs7961152 | 570 | 963 | 416 | 992 | 1448 | 492 | 19.54 | 20.09 | -0.027 | Add |
| rs1937506 | 1097 | 742 | 113 | 1484 | 1205 | 244 | 18.17 | 19.66 | -0.076 | Add |
| rs6997709 | 1116 | 716 | 118 | 1500 | 1201 | 237 | 19.86 | 19.97 | -0.005 | Add |

*Data were extracted from Loley et al. (2013)

$r_{i}$, $s_{i}$: genotype counts for each SNP. $\Delta T$: $\left( T_{JT}-T_{CA}^{Add} \right)/{T_{CA}^{Add}}$. Model: Plausible genetic model inferred from genotype counts
