# Supplementary material for: A nonparametric alternative to the Cochran-Armitage trend test in genetic case-control association studies: The Jonckheere-Terpstra trend test
Source: PLoS One. 2023 Feb 2;18(2):e0280809. doi: 10.1371/journal.pone.0280809 (PMC9894441; doi:10.1371/journal.pone.0280809)
Supplement: S3 Table — (DOCX) [file pone.0280809.s003.docx]

**Table S3: Exact p-values of the Hardy-Weinberg equilibrium (HWE) tests among cases and controls of the variants and the allelic test statistics in the Real data analysis**

| SNP | Study | P-value: Cases | P-value: Controls | Allelic test: statistic (P-value) |
| --- | --- | --- | --- | --- |
| rs2398162 | Hypertension | 0.013 | 0.961 | 20.2 (7.0e-6) |
| rs10900589 | Falciparum malaria (Ghanaian) | 0.082 | 0.028 | 13.3 (2.7e-4) |
| rs380390 | Falciparum malaria (Ghanaian) | 0.224 | 0.765 | 28.8 (8.1e-8) |
| rs10900589 | Falciparum malaria (Gambian) | 0.180 | 0.068 | 1.3 (0.26) |
| rs10131337 | Falciparum malaria (Gambian) | 1.000 | 0.134 | 21.9 (2.8e-6) |
| rs7961152 | Hypertension | 0.820 | 0.361 | 19.8 (8.6e-6) |
| rs1937506 | Hypertension | 0.430 | 1.000 | 19.3 (1.1e-5) |
| rs6997709 | Hypertension | 0.806 | 0.892 | 19.8 (8.6e-6) |
