# Supplementary material for: A nonparametric alternative to the Cochran-Armitage trend test in genetic case-control association studies: The Jonckheere-Terpstra trend test
Source: PLoS One. 2023 Feb 2;18(2):e0280809. doi: 10.1371/journal.pone.0280809 (PMC9894441; doi:10.1371/journal.pone.0280809)
Supplement: Attachment — Submitted filename: Reviewer-Points-Comments.docx [file pone.0280809.s004.docx]

**Dear Editor-in-Chief**

**Plos One Journal**

I have reviewed the manuscript entitled: "**nonparametric alternative to the Cochran-Armitage trend test in genetic case-control association studies: The Jonckheere-Terpstra trend test**", with Manuscript Number: **PONE-D-21-39215**. This manuscript is an interesting topic that could help researchers to tackle any bias results might be happened in the related analysis. But still need more revisions (major/minor) that I have mentioned viewpoints/ comments as below:

**# Abstract:**

Abstract is too general, while it would be better to be more specific in case of the definition and importance of the problem, and authors should give evidences in which are supported with quantitative data such as sample size etc.!

**#Introduction:**

In Line 47: it seems that sentence is incomplete ...is not additive (e.g.,(Gonzalez et al., 2008;…

Generally: This section at this format seems to be non-informative, authors must give more evidences of reported studies, try to highlight the weakness including suffering from the power loss etc., then discuss importance and objectives of the method of interest with more details.

**# Simulation:**

In Lines 113~120: authors must present more supportive data in case of sample size and MAFs to show advantages/ dis-advantages of both methods in analyzing genome-wide association studies.

**# Real Data Analysis:**

At this section, authors are strongly advised to use more real data set for comparing the efficiency of the two tests. With few cases, we are not able to observe power/failure of each method.

**# Tables:**

In table 2: Could authors show whether the calculated statistics at different sample size are significantly different? By the way, the table was not referred in the main text.

With the best regards

**Ali Moumeni**
