# Supplementary material for: A nonparametric alternative to the Cochran-Armitage trend test in genetic case-control association studies: The Jonckheere-Terpstra trend test
Source: PLoS One. 2023 Feb 2;18(2):e0280809. doi: 10.1371/journal.pone.0280809 (PMC9894441; doi:10.1371/journal.pone.0280809)
Supplement: Attachment — Submitted filename: Plos One response letter.docx [file pone.0280809.s005.docx]

Point-by-point response to comments from Reviewer

Thank you very much for reviewing our paper and the detailed and helpful review report. We greatly appreciate your time, effort, encouragement, and insight. We have revised our paper addressing all issues raised in your report. The following is our point-by-point response to your comments. For convenience, your original comments are copied and our replies follow in blue. The associated revisions in the manuscript are highlighted in track change.

Comments:

I have reviewed the manuscript entitled: "nonparametric alternative to the Cochran-Armitage trend test in genetic case-control association studies: The Jonckheere-Terpstra trend test", with Manuscript Number: PONE-D-21-39215. This manuscript is an interesting topic that could help researchers to tackle any bias results might be happened in the related analysis. But still need more revisions (major/minor) that I have mentioned viewpoints/ comments as below:

Thank you very much for your concise summary and encouraging comment. We appreciate very much for your time and effort.

\# Abstract:

Abstract is too general, while it would be better to be more specific in case of the definition and importance of the problem, and authors should give evidences in which are supported with quantitative data such as sample size etc.!

Thank you for the comment. In this revision, we have provided more related materials and details in the Abstract.

#Introduction:

In Line 47: it seems that sentence is incomplete ...is not additive (e.g.,(Gonzalez et al., 2008;…

In this sentence, the period is after the long list of citations, so it may appear that the sentence is incomplete:

*However, it can suffer from power loss when the true genetic model is not additive (e.g., Gonzalez et al., 2008; Kuo & Feingold, 2010; Li, Zheng, Liang, & Yu, 2009; Loley, König, Hothorn, & Ziegler, 2013).*

When reviewing it, however, we did find an extra “(” after “e.g.,”, and we have removed it in this revision (line 55, page 4).

Generally: This section at this format seems to be non-informative, authors must give more evidences of reported studies, try to highlight the weakness including suffering from the power loss etc., then discuss importance and objectives of the method of interest with more details.

We appreciate this suggestion to add more details in Introduction. In this revision, we expanded Introduction by providing more context information and details for the tests and significance of the research question.

\# Simulation:

In Lines 113~120: authors must present more supportive data in case of sample size and MAFs to show advantages/ dis-advantages of both methods in analyzing genome-wide association studies.

In the original simulation, we considered sample sizes of $N\in\left( 200, 500, 1000 \right)$ and MAFs of $q\in\left( 0.05, 0.1,0.2,0.3 \right)$. To evaluate the relative performance of the CA and JT trend tests in a wider range of data scenarios, we further considered the sample size of $N=1500$ and $2000$ as well as $q=0.4$. Because the sample sizes and MAF were fairly large, we reduced the effect size in the alternative hypothesis to $\lambda=0.5$ to make the power comparison meaningful (otherwise the power of both tests will be close to 1 and hence difficult to evaluate the relative performance).

The empirical power of the two tests for the additional simulation settings were summarized in Table S1 in the Supplementary Materials. The results were consistent with original conclusion: compared to the CA trend test, the JT trend test is more powerful when the underlying genetic model is dominant. The power advantage of $T_{JT}$ diminishes as the genetic model evolves toward the additive model and have approximately equivalent power when the underlying model is additive. $T_{JT}$ becomes less powerful than $T_{CA}^{Add}$ and the disadvantage enlarges when the genetic model keeps evolving toward the recessive end.

\# Real Data Analysis:

At this section, authors are strongly advised to use more real data set for comparing the efficiency of the two tests. With few cases, we are not able to observe power/failure of each method.

Thank you for the suggestion. In this revision, we compared the two tests on additional studies for SNPs that were reported associated with hypertension. The results were reported in Table S2 in the Supplementary Materials. The conclusion still holds in this real data analysis: $T_{JT}$ and $T_{CA}^{Add}$ had similar power when the genetic model tended to be additive (rs7961152, rs1937506, rs6997709) and $T_{JT}$ is more powerful than $T_{CA}^{Add}$ when the genetic model tended to be dominant (rs2398162).

\# Tables:

In table 2: Could authors show whether the calculated statistics at different sample size are significantly different? By the way, the table was not referred in the main text.

Thank you for the comment and we apologize for not referring Table 2 in the main text in the original submission. This table refers to the simulation findings verification using expected theoretical tables for each simulation setting (last paragraph of Simulation section) and we now explained Table 2 in the right place of the manuscript.

We would like to explain that the test statistics ($T_{JT}$ and $T_{CA}^{Add}$) in Table 2 were calculated based on “expected theoretical tables”. Specifically, in each simulation setting, using the fixed combination of sample size, MAF, and genetic model, the cell probabilities of each genotype for cases and controls in the genotype distribution table (Table 1) can be calculated, and therefore, the expected cell values can also be calculated by multiplying the probabilities with the sample size. We refer such table consisted of the expected cell values as a “expected” table, which allows us to evaluate the relative performance of the two tests by comparing their theoretical test statistics ($T_{JT}$ and $T_{CA}^{Add}$) in each simulation setting. The relative difference between theoretical test statistics ($\Delta T=\frac{\left( T_{JT}-T_{CA}^{Add} \right)}{T_{CA}^{Add}}\times100\%$) are then reported in Table 2, which are used to verify the empirical findings observed in simulation. To fully explain this, we also provided more details and clarifications to this paragraph in this revision. However, since each simulation setting corresponds to a single $\Delta T$ that is non-random, we cannot assess the statistical significance of $\Delta T$. Instead, readers may refer to Figure 1 for the actual power comparison from simulation, which can serve as a proxy for the relative statistical significance between the two tests.
