# Supplementary material for: A nonparametric alternative to the Cochran-Armitage trend test in genetic case-control association studies: The Jonckheere-Terpstra trend test
Source: PLoS One. 2023 Feb 2;18(2):e0280809. doi: 10.1371/journal.pone.0280809 (PMC9894441; doi:10.1371/journal.pone.0280809)
Supplement: Attachment — Submitted filename: Response letter.docx [file pone.0280809.s007.docx]

**Reviewer #2: This article concerns a power comparison of the Cochran-Armitage trend test and the non-parametric Jonckheere-Terpstra trend test, under common genetic models (additive, dominant, recessive), different minor allele frequencies and sample sizes, and for bi-allelic genetic variants. The article is concise and well written, and the conclusions are clear. I have some major and minor concerns detailed below.**

Thank you very much for your concise summary and encouraging comment. We appreciate very much for your time and effort.

**Major concerns:**

**The example in the Real data analysis section on page 9 is not clearly a case of dominance; in fact, a co-dominant model seems to be the best, where the heterozygote has the highest risk. Variant rs20541 is thus a poor example to make the case of an advantage of the Jonckheere-Terpstra test. In fact, the clearest case of dominance is rs2398162 in Table S2. If the authors wish to practically illustrate the advantage of the Jonckheere-Terpstra test, then rs2398162 would be a better choice.**

Thank you for this suggestion. In this revision we updated this section by using the variant rs2398162 in the hypertension study in the Real data analysis as an example of dominant model. Relevant text and Table S2 were also updated to reflect this change.

**On line 192 the authors state “variant rs10900589 in ATP2B4 was associated with the disease in the Ghanaian samples and the association was replicated in the Gambian samples”. This statement is obviously FALSE. For Figure 2 shows very significant association for the Ghanaian sample, but a clearly non-significant association for the Gambian sample. Please correct the sentence.**

Thank you for this observation and we apologize that the previous statement was inaccurate. In Loley et al. (2013, EJHG 21:1442-1448) it was shown this signal was only significant when coded in a recessive model in the Gambian group (Table 1). In this revision we corrected the sentence and stated that “*This association was also evaluated in the Gambian samples and it was significant under a recessive model but insignificant under dominant and additive models.*”

**In genetic association studies it is common to test the SNPs involved for Hardy-Weinberg equilibrium. If there are significant deviations from HWE, the results of the association tests may be questioned, because disequilibrium is potentially indicative of the presence of genotyping errors (Hosking et al, 2004; Leal, 2005). The HWE testing can be done with many genetic data analysis software such as PLINK (Purcell et al., 2007) or R-package Hardy-Weinberg (Graffelman, 2015). It is recommended to test cases and controls separately, and report exact Hardy-Weinberg p-values for each group. Exact testing is the preferred approach, for it has the highest power. I suggest the authors to include HW test results in the paper, for this can only improve the credibility of their conclusions.**

Thank you for this suggestion. In the revision we conducted the exact test for HWE among the cases and controls for each of the variant using the suggested R package (HardyWeinberg). The p-value results were summarized in S3 Table and we concluded that “*Results showed that the p-values of the HWE tests for all the variants were larger than 0.01, with only two between 0.01 and 0.05, suggesting that there was little evidence of genotyping error among the variants*”.

**The authors emphasize the power gain of the Jonckheere-Terpstra test under the dominant model. However, the power loss of this test under recessive model is generally much larger than the gain under the dominant model (see Figure 1, Table 2). In practice one often does not know the correct genetic model. One thus say that, a priori, and overall, the Cochran Armitage test may be the best choice, at least if the MAF is not low. This conclusion should be added to the evaluation of the tests in the Discussion section**

Thank you for bringing up this excellent point. In this revision, we provided such information about the selection between JT and CA trend tests when the true genetic model is unknown at the end of the discussion.

**In genetic association studies with SNPs, it is also common to test for association not only at the genotype level, but also at the level of alleles. A standard test for this purpose is the so called alleles test (Laird & Lange, 2011). For all empirical 9 SNPs (Page Figure 2, Table S2) the alleles test leads to the same conclusion as the Jonckheere-Terpstra test. This could at least be stated, as it strengthens the conclusions of the “Real data analysis” Section.**

Thank you for this suggestion. We conducted the allelic test for the variants included in the Real data analysis section and confirmed that the test results were close to the discussed methods. The results of the allelic test were included in S3 table.

**Minor issues:**

**L59: “recource” -- “resource”**

**L138: “set of simulation” -- “set of simulations”**

**L161: “We refer this table consisted of” -- “This table consists of”**

**L162: delete “as an expected table”.**

**L167, L202: “were reported” -- “are reported”**

**L262: capitalize “Kendall”**

Thank you for these observations. We have made corresponding changes in this revision.
